# Hexakis[p-(hydroxymethyl)phenoxy]cyclotriphosphazene as an Environmentally Friendly Modifier for Polyurethane Powder Coatings with Increased Thermal Stability and Corrosion Resistance

**DOI:** 10.3390/ma17112672

**Published:** 2024-06-01

**Authors:** Barbara Pilch-Pitera, Dominika Czachor-Jadacka, Łukasz Byczyński, Michał Dutkiewicz, Rafał Januszewski, Krzysztof Kowalczyk, Wojciech J. Nowak, Katarzyna Pojnar

**Affiliations:** 1 Faculty of Chemistry, Department of Polymers and Biopolymers, Rzeszow University of Technology, al. Powstańców Warszawy 6, 35-959 Rzeszow, Poland; d.czachor94@gmail.com (D.C.-J.); lbyczynski@prz.edu.pl (Ł.B.); 2 Poznan Science and Technology Park, Adam Mickiewicz University Foundation, Rubież 46, 61-612 Poznan, Poland; midu@ppnt.poznan.pl; 3 Faculty of Chemistry, Adam Mickiewicz University in Poznan, Umultowska 89B, ul. Uniwersytetu Poznańskiego 8, 61-614 Poznan, Poland; r.janusz@amu.edu.pl; 4 Center for Advanced Technology, Adam Mickiewicz University in Poznan, ul. Uniwersytetu Poznańskiego 10, 61-614 Poznan, Poland; 5 Faculty of Chemical Technology and Engineering, Polymer Institute, West Pomeranian University of Technology in Szczecin, ul. Pułaskiego 10, 70-322 Szczecin, Poland; krzysztof.kowalczyk@zut.edu.pl; 6 Faculty of Mechanical Engineering and Aeronautics, Department of Materials Science, Rzeszow University of Technology, al. Powstańców Warszawy 8, 35-959 Rzeszow, Poland; wjnowak@prz.edu.pl; 7 Doctoral School of Engineering and Technical Sciences at the Rzeszow University of Technology, al. Powstańców Warszawy 12, 35-959 Rzeszow, Poland; d521@stud.prz.edu.pl

**Keywords:** hexakis(4-(hydroxymethyl)phenoxy)cyclotriphosphazene, powder coatings, anticorrosion protection, thermal stability

## Abstract

Protection against fire and the corrosion of metals is necessary to ensure human safety. Most of the fire and corrosion inhibitors do not meet the ecological requirements. Therefore, effective and ecological methods of protecting metals are currently a challenge for researchers. In this work, the influence of hexakis(4-(hydroxymethyl)phenoxy)cyclotriphosphazene (HHPCP) on the characteristics of powder coatings was examined. The coatings’ properties were investigated by measuring the roughness, hardness, adhesion to the steel surface, cupping, gloss, scratch resistance, and water contact angle. The thermal stability was studied by furnace test and TGA analysis. The corrosion resistance test was carried out in a 3.5% NaCl solution. The distribution of phosphazene-derived segments in the coating was examined by GD-EOS analysis. Modified coatings show better corrosion and thermal resistance and can be used for the protection of the steel surface. Their better corrosion resistance is due to the electroactive properties of the phosphazene ring and its higher concentration at the coating surface, confirmed by GD-EOS analysis. The increase in thermal resistance is due to the effect of the formation of phosphoric metaphosphoric and polyphosphoric acids during the decomposition of HHCPC, which remain in the condensed char phase and play a crucial role in surface protection.

## 1. Introduction

In recent years, there has been a growing interest in environmentally friendly coatings that still provide various functionalities and satisfy consumer requirements [1,2]. Powder coatings (PCs) are a better alternative to conventional liquid products because of the multitude of advantages resulting from the lack of organic solvents in their composition. For these reasons, PCs meet requirements related to the minimization of volatile organic compound (VOC) emissions. Compared to liquid paints, powder coatings are defined by, e.g., a short time to obtain the finished painting (as opposed to liquid paints, the PC is ready for use after taking it out of the oven with no need to wait for it to dry), lower costs of waste disposal (less waste due to the possibility of reusing the powder falling from the detail during spray), and durability (excellent properties of the final product). Powder coatings offer a wide range of modifications. According to the latest reports, the conductive properties [3,4,5,6], antibacterial [7], amphiphobic [8], and wear resistance [9,10] are described. 

This work focuses on the application of cyclotriphosphazene to modify polyurethane powder coatings in order to improve their properties, such as fire resistance or corrosion protection. Flame-retardant coatings are in high demand in the automotive, aviation, and construction industries. Flame retardancy of powder coating is particularly desirable to reduce fire damage and also provide time for the safe evacuation of people [11,12,13]. Phosphorus compounds, together with boron compounds, are commonly reported types of coating modifiers with decreased flammability [14]. The general mechanism of anti-flammable materials depends on the flame retardant given and the type of substrate [15]. Flame-retardant additives can delay combustion in both the gas phase through flame inhibition and condensed phases (e.g., polymer matrix) by enhancing charring. Efficiency depends not only on the flame retardant itself but also on its interaction with the coating binder and additives. The coating surface can undergo charring, which creates a thermal insulation barrier between the burned and unburned parts. The created barrier slows the heat transfer to the unburned part. Active additives in the gas phase also provide an increase in the protection of flammable materials, but the release of hydrogen chloride or hydrogen bromide is damaging to our health and the environment, and its usage has been restricted [16]. In addition, a fire retardant is a substance that acts by physical or chemical action, reducing the flammability of materials or causing delays in their combustion. In the physical mechanism, a protective coating can be created to lower the ignition temperature. There are also many ways to increase the flame retardancy of polyurethane. Chattopadhyay et al. described reports on the use of such compounds as flame retardants, including halogenated paraffin, chlorofluorocarbons, inorganic oxides, and hydroxides (e.g., antimony oxide, magnesium hydroxide, aluminum hydroxide), inorganic carbonates (e.g., potassium, calcium, magnesium carbonate), inorganic boron compounds (e.g., boric acid, zinc borate borax), phosphorus compounds, expanded graphite, melamine, mica, and organic compounds of boron and silicon. The addition of expanded graphite dyes the coating black, which limits its dyeing to bright colors. Compounds containing halogen atoms, during a fire, release poisonous and asphyxiating hydrogen halides that destroy the ozone layer. Furthermore, when they are burning, some additives can release poisonous carbon monoxide (CO) [17]. The application of fire-retardant coatings is among the simplest and most effective ways to prevent fire in materials. This approach presents several advantages, allowing easy processing and no modification of the mechanical properties of the protected material. Among the most promising halogen-free solutions are intumescent flame-retardant (IFR) coatings. They consist of three active ingredients: an acidic catalyst (e.g., ammonium polyphosphate), a charring agent (e.g., polyol), and a blowing agent (most commonly ammonium compounds or melamine) connected by a polymeric binder. As a result of heat exposure, the intumescent coating swells, increasing many times in its original thickness, and produces a foamed carbonaceous char, which acts as an insulating layer to protect the substrate [18]. However, an effective interaction of intumescent components requires a coating thickness of more than 300–500 µm [19]. In the case of powder coatings with such high thickness, their properties are significantly deteriorated. According to technical requirements, e.g., Qualicoat [20], the optimal thickness of a single powder coating should be 60 µm.

The discovery of the cyclotrifosphazene compound by Allock and his colleagues in the mid-1950s allowed for relatively mild combustion properties, the reduced production of toxic gases, and its use as a flame retardant [21]. Due to the high reactivity of cyclotriphosphazene compounds, it is possible to introduce a wide range of substituents through nucleophilic reactions. Consequently, various side groups affect the characteristics of polyphosphazenes. In their review, Usri et al. discussed research on cyclotriphosphazene, with concentration on the synthesis, structural characteristics, dielectric characteristics, and flame retardancy of the compound hexachlorocyclotri-phosphazene.

Many polymer materials are characterized by good chemical, physical, and mechanical properties. But it is hard to find a material that can be characterized by specific properties, such as fire resistance or corrosion protection. Zhou et al. described the synthesis, curing kinetics, thermal properties, and flame retardancy of cyclotriphosphazene-containing multifunctional epoxy resin. For its modification, the author used the following commercial products: DGEBA (diglycidyl ether of bisphenol A) and hexachlorocyclotriphosphazene (HCCP). Multifunctional epoxy resin hexa-[4-(glycidyloxymethyl)phenoxy]-cyclotriphosphazene (HGPCP) cured with 4,4′-diaminodiphenyl methane (DDM) and 4,4′-diaminodiphenyl sulfone (DDS) exhibited excellent flame retardancy during the test via the limiting oxygen index (LOI) and vertical burning test (UL-94) [22]. Xu et al. also investigated flame-retardant properties based on epoxy resin. The LOI test and horizontal flame test showed that the presence of hexakis[p-(hydroxymethyl)phenoxy]cyclotriphosphazene (HHPCP) also leads to improved flame retardancy properties of epoxy resin [23]. Epoxidized hexakis-4-(2-(4-((β-methallyl)oxy)phenyl)propan-2-yl)phenoxycyclotriphosphazene by 3-chloroperbenzoic acid was cured by its treatment with isophorone diamine, which characterized improved thermal stability and glass transition temperature [24]. In addition to fire and heat resistance, epoxy resin based on arylaminocyclotriphosphazene improves chemical and corrosion resistance, high tensile strength, adhesion, and low solidification [25]. Consequently, these substances find applications in numerous cutting-edge scientific and technological domains, including coatings, adhesives, composites, extractants, metal complexes, organometallic chemistry, inorganic chemistry, aviation, aerospace, radioengineering, nuclear, and medical fields [26,27]. 

Improvements in fire resistance were noted even with the application of cyclotriphosphazene to combustible polypropylene. Flame-retardant polypropylene (PP) was obtained by appropriately blending with microencapsulated ammonium polyphosphate (MAPP). The modified polypropylene was characterized by an increase in the oxygen index (LOI) [28]. Mohd Taip and al. described the increase in the LOI value in the 2K polyurethane wood coating containing hexasubstituted cyclotriphosphazene compounds [29].

Generally, cyclotriphosphazene has often been used as a modifier and as a flame retardant in many polymers. Nevertheless, based on our analysis of the relevant research, there are no reports of modifications of powder coatings with the cyclotriphosphazene compound. 

Corrosion is also one of the main challenges facing metal component manufacturers. The duration of corrosion protection depends on the high adhesion to the surface, tightness, and resistance of the coating to the corrosive environment. Powder coatings perfectly protect, stop, or slow down the spontaneous corrosion process. Corrosion resistance is an important element in the durability and aesthetics of objects. Currently, the most commonly used corrosion protection is metallic coatings, e.g., Zn or Cr [30,31,32] and polymer coatings [33]. However, the use of Cr and Zn is limited due to environmental and economic considerations. The alternative is powder coatings, which can be modified to obtain the appropriate properties, for example, excellent adhesion to the substrate surface, resistance to atmospheric environments, and hydrophobic properties [34]. The hydrophobic coating repels moisture and reduces the absorption of water and corrosive media to the surface of the coatings. However, in a highly corrosive environment, for more effective protection, the barrier made of a polymer binder may be insufficient, and the addition of corrosion inhibitors is necessary. The anticorrosion protection of powder coatings can be improved using various types of additives, e.g., ceramic powders, pigments, and fillers [35]. An example is the invention described in CN106189682A, which reveals the use of intercalated montmorillonite for the modification of epoxy resin-based powder coatings for the protection of pipeline corrosion [36]. Similarly, the addition of electroactive conductive polymers, such as polyaniline or polypyrrole, may increase the protection of steel in acidic or neutral environments by the repassivation of exposed areas [37]. Cyclophosphazene derivatives contain P heteroatoms and may also be applicable as corrosion inhibitors on metal surfaces for their treatment/protection against the corrosive environment [26]. However, polyurethane high-solid coatings show a decrease in hydrophobicity after modification with epoxyfunctional cyclotriphosphazene containing bulky aromatic substituents and silicon chains [38]. 

Moreover, the influence of functionalized cyclotriphosphazene on the properties of polyuretane powder coatings has not been studied so far. For this reason, the novelty of this work is the use of hexakis(4-(hydroxymethyl)phenoxy)cyclotriphosphazene as a reactive modifier of polyurethane powder coatings and the examination of its effect on coating properties.

The scope of this research was to investigate the influence of the hexakis(4-(hydroxymethyl)phenoxy)cyclotriphosphazene modifier on the properties of powder coatings based on polyester resin and the polyisocyanate hardener. The anticorrosion protection of the coatings, gloss, water contact angle, roughness, cupping, hardness, adhesion, and scratch resistance of the coatings was examined. The coating’s thermal properties were tested using the thermogravimetric method (TGA). Additionally, fire protection properties were examined using the furnace test method. 

## 2. Experimental Section

### 2.1. Materials

Hexachlorocyclotriphosphazene (HC) and 4-hydroxybenzaldehyde were purchased from Sigma Aldrich (Darmstadt, Germany). Potassium carbonate_,_ tetrahydrofuran (THF), sodium tetrahydroborate (NaBH_4_), dichloromethane, ethanol, and methanol were purchased from Merck (Darmstadt, Germany).

Sirales PE 6110—hydroxyl functionalized saturated polyester resin containing isophthalic acid as well as neopentyl glycol (acid value: 4–8 mg KOH/g, hydroxyl value: 30–45 mg KOH/g) was supplied by Sir Industriale (Macherio, Italy). A blocked Vestagon B1530 polyisocyanate, which contained 14.8–15.7% of the isocyanate group, was delivered from Evonik Industries (Marl, Germany). A Resiflow PV 88 flow control agent was provided by Worlèe Chemie GmbH (Lauenburg, Germany), and benzoin (degassing agent) was supplied from Sigma Aldrich (Darmstadt, Germany). 

### 2.2. Synthesis of Hexakis(4-(hydroxymethyl)phenoxy)cyclotriphosphazene (HHPCP)

Hexakis(4-(hydroxymethyl)phenoxy)cyclotriphosphazene (3) was obtained in a two-step nucleophilic substitution reaction of Cl atoms in hexachlorocyclotriphosphazene (1) with 4-hydroxybenzaldehyde in the presence of potassium carbonate, leading to the formation of hexakis(4-formylphenoxy)cyclotriphosphazene (2) in the first step and a further reduction in the aldehyde groups present in (2) to the hydroxyl ones carried out in the presence of sodium borohydride in the second step.

**Scheme 1:**
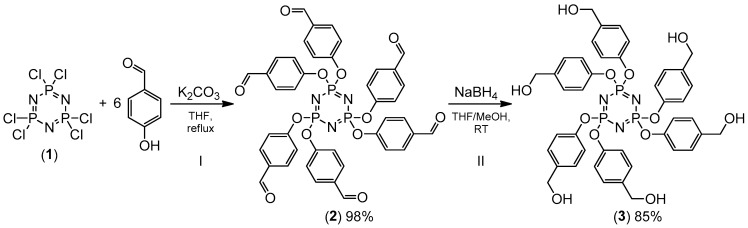
Synthetic path for hexakis(4-(hydroxymethyl)phenoxy)cyclotriphosphazene (3) from hexachlorocyclotriphosphazene (1) through the formation of hexakis(4-formylphenoxy)cyclotriphosphazene (2).

Based on the synthetic path illustrated in Scheme 1, in the first step, hexachlorocyclotriphosphazene (1) (5 g, 14.38 mmol), K_2_CO_3_ (28.52 g, 206.35 mmol), and 4-hydroxybenzaldehyde (10.54 g, 86.3 mmol) in THF (150 mL) as a solvent were placed together in a 250 mL round-bottom flask with a gas funnel and condenser attached. The reaction mixture was then refluxed under an Ar atmosphere until the complete conversion of substrates was monitored with FT-IR spectroscopy. The solvent was then evaporated, and the product was extracted three times with dichloromethane from the residue. Hexakis(4-formylphenoxy)cyclotriphosphazene (2) as a crystalline solid was isolated by vacuum dichloromethane evaporation (98.60% yield) and subjected to FT-IR, ^1^H, ^13^C, and ^31^P NMR analysis, which confirmed its structure [^1^H NMR (CDCl_3_): 7.13 (d, 12H), 7.72 (d, 12H), 9.91 (s, 6H) ppm; ^13^C NMR (CDCl_3_): 121.29, 131.46, 133.83, 154.56, 190.50 ppm; ^31^P NMR (CDCl_3_): 7.09 ppm]. The spectroscopic analysis results for the hexakis(4-formylphenoxy)cyclotriphosphazene (2) are consistent with data from the literature [23]. The FT-IR, ^1^H, ^13^C, and ^31^P NMR spectra of hexakis(4-formylphenoxy)cyclotriphosphazene (2) are presented in Figures S1–S4 (see Supplementary Materials).

**Figure 1:**
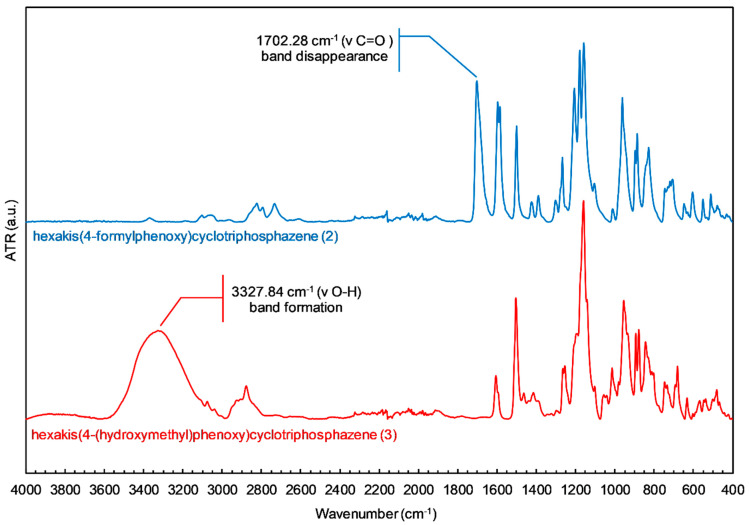
Stacked FT-IR spectra of hexakis(4-formylphenoxy)cyclotriphosphazene (2) as a substrate and hexakis(4-(hydroxymethyl)phenoxy)cyclotriphosphazene (3) as a product.

In the second step, obtained hexakis(4-formylphenoxy)cyclotriphosphazene (2) (10 g, 11.6 mmol) was placed in a 250 mL round-bottom flask equipped with a condenser and gas funnel and dissolved in 200 mL of a THF/MeOH (1:1) mixture. Next, NaBH_4_ (2.85 g, 75.31 mmol) was slowly added to the phosphazene (2) solution in RT (room temperature). The reaction was carried out in RT to complete the conversion of CHO groups (evaluated based on the band intensity at 1702 cm^−1^ on the FT-IR spectra of the reaction mixture in Figure 1). After the completion of the reaction and solvent evaporation, the obtained crude product was dissolved in EtOH, precipitated with water, filtered, and washed three times with water. Dried at 80 °C, hexakis(4-(hydroxymethyl)phenoxy)cyclotriphosphazene (3) as a white powder (85.3% yield) was subjected to the FT-IR, ^1^H, ^13^C, and ^31^P-NMR analysis which confirmed its structure [^1^H NMR (DMSO-d6): 4.47 (d, 12H), 5.23 (t, 6H), 6.80 (d, 12H) 7.20 (d, 12H) ppm; ^13^C NMR (DMSO-d6): 62.28, 120.13, 127.70, 139.45, 148.62 ppm; ^31^P NMR (DMSO-d6): 8.86 ppm]. The spectroscopic analysis results for hexakis(4-(hydroxymethyl)phenoxy)cyclotriphosphazene (3) are consistent with data from the literature [23]. The FT-IR, ^1^H, ^13^C, and ^31^P NMR spectra of hexakis(4-(hydroxymethyl)phenoxy)cyclotriphosphazene (3) are presented in Figures S5–S8 (see Supplementary Materials).

### 2.3. Method of Preparation of Powder Coatings

The formulations of polyurethane powder coatings included a blocked polyisocyanate Vestagon B1530 and a polyester resin PE6110 in the molar ratio of the -NCO:-OH group equal to 1:1, benzoin as degassing agents (0.25 wt%), Resiflow PV 88 as the leveling agent (0.5 wt%), which consists of a liquid acrylic polymer that has been adsorbed onto a silica filler, as well as hexakis(4-(hydroxymethyl)phenoxy)cyclotriphosphazene as a modifier (1.5 wt%, 2.5 wt% and 5 wt%). The reference sample does not contain the modifier. The PC formulation was milled and extruded in a corotating twin screw mini extruder (EHP 2 × 12 Sline from Zamak (Poland). The temp. distribution in the extruder was as follows: zone I—90 °C, zone II—120 °C, zone III—122 °C, and adapter—125 °C. The screw rotational speed was 120 r.p.m. After extrusion, the mixture was cooled, crushed, pulverized, and subsequently sieved using a 100 μm sieve. Using an electrostatic gun, PEM X-1, controlled by EPG Sprint X (CORONA) from Wagner (Altstätten, Switzerland), the final powder coatings were applied to the steel panels made of DC01 stamping steel, cold-rolled, 0.5 mm thick, which were previously cleaned and prepared. The electrode voltage was 30 kV. The powder particles in the gun were electrified by an electrode positioned in the nozzle after being transported from the tank by compressed air. The charged particles were then transferred to ground steel plates. To prepare the surface, the steel plates were first degreased with acetone and then immersed in a 1.5% aqueous solution of ESKAPHOR Z 2000C, pH = 5.5 at a temperature of 30 °C for 2 min to apply the zirconium phosphate conversion coating. The panels were then rinsed with distilled water and dried after the water was removed. The coatings were then cured in an oven at 220 °C for 15 min. The names given to the cured coatings were based on the modifier content; for example, PU/1.5%Ph means polyurethane coating with a 1.5% cyclophosphazene modifier (Table 1). 

### 2.4. Coatings Characterization

#### 2.4.1. Polymerization Test

The polymerization test was carried out in accordance with Qualicoat specifications [20]. A cotton wool swab was saturated with the MEKO solvent. Within 30 s, the powder coating was lightly rubbed 30 times in each direction. After 30 min, the polymerization quality was evaluated in accordance with the following ratings:

1. The coating is quite soft and matt;
2. The coating is matt and can be scratched with a fingernail;
3. Gloss loss is less than 5 units;
4. No noticeable changes. The coating cannot be scratched with a fingernail.

#### 2.4.2. Glow Discharge Optical Emission Spectrometry (GD-EOS)

Glow discharge optical emission spectroscopy analysis (GD-OES) was performed on coatings with a different amount of the phosphazene modifier on the steel substrate. The GD-OES depth profiles were achieved using a GD Profiler HR (Horiba Jobin Yvon). All analyses were conducted under the same sputtering conditions, that is, with an Ar pressure of 700 Pa and 30 W power. A copper anode of 4 mm in diameter was used. The analysis used Ar with a very high purity of 6 N. As a result of the different content of the phosphazene modifier, the phosphorus profiles of the coatings were analyzed. A higher light intensity corresponded to a higher concentration of measured elements at a certain depth. The wavelength of 178 nm was used for light detection emitted by phosphorous.

#### 2.4.3. Performance Properties

A Mar Surf PSI profilometer (Mahr GmbH, Göttingen, Germany) was used to measure the roughness of the coating in accordance with the ISO 12085 [39] standard. The measuring needle was positioned on the cured coating, and the profilometer was leveled. The device then automatically conducted the measurement by moving the needle along the surface of the coating. Measurements were taken at multiple locations on the coating. Ten coated panels were tested, and the arithmetic mean was calculated. The roughness value was determined using the Ra parameter (arithmetic mean of the roughness profile deviated from the baseline) and the Rz parameter (arithmetic mean of the five highest-profile hills decreased by the arithmetic mean of the five lowest-profile depths).

The coating gloss and thickness were measured using a micro-TRI-gloss µ gloss meter from Byk-Gardner (Geretsried, Germany) in accordance with the ISO 2813 [40] (gloss) and ISO 2808 [41] (thickness) standard. Ten coated panels were tested, and the arithmetic mean was calculated.

The surface adhesion of the coatings was assessed by a cross-cut test in accordance with the ISO 2409 [42] standard. Six cutters knife from Byk-Gardner (Geretsried, Germany) was used to make a cut through the coating into the substrate. The surface of the incision network was examined with the naked eye and classified on a six-point scale from 0 to 5. The best surface adhesion was observed in coatings classified with a score of 0, where the edges of the incision were completely smooth. Conversely, the worst adhesion was marked with a score of 5, where the damaged surface of the incision network exceeded 65%. Measurements were taken at 3 coated panels of each composition, and the arithmetic mean was calculated.

The relative hardness of the cured powder coatings was examined using the König Pendulum tester from BYK-Gardner (Geretsried, Germany) in accordance with the ISO 1522 [43]. The relative hardness value was determined as the ratio of the damping time of an oscillating pendulum supported on the coating surface to the time noted for the pendulum supported on a glass plate. The test was conducted in three locations on the coated steel plates, with two plates of each composition examined, resulting in six measurements for each sample; then, the arithmetic mean was calculated.

The cupping of the cured powder coatings was evaluated by a manual cupping tester in accordance with the ISO 1520 [44]. Cupping is the minimum depth, expressed in millimeters, at which the coating is damaged during mechanical deformation.

The scratch resistance was determined using the Elcometer Clemen Tester (Manchester, UK) in accordance with ISO 1518-1 [45]. Three coated panels were tested for each composition. The measurement involved determining the minimum load required to scratch the coating using a needle with a semicircular tip.

#### 2.4.4. Water Contact Angle

The water contact angle was measured by the ‘sitting drop’ method using an OCA15 EC optical goniometer by Data Physics according to the EN 828 [46] standard. The drop volume was 1 μL. The measurement temperature was 24 ± 1 °C. The drop images were captured with a camera. The contact angle was determined using a control program, following the prior establishment of the baseline and drop contour. The arithmetic mean of 10 measurements was taken as the final result.

#### 2.4.5. Thermogravimetric Analysis

Thermogravimetric analysis was carried out using a Mettler Toledo TGA/DSC1 instrument (Mettler Toledo, Columbus, OH, USA). The measurements were performed in a nitrogen atmosphere in the temperature range of 25 to 700 °C at a heating rate of 10 °C/min. The sample weight was ~5 mg, the gas flow rate was 50 cm^3^/min, and a 150 μL open alumina pan was used.

#### 2.4.6. Furnace Tests

A gas flame-heated programmable laboratory furnace, equipped with two slots for steel plates coated with the tested material, was used to measure the thermal insulation properties of the prepared intumescent coatings. During the furnace test, two coated steel samples (ca. 10 × 10 cm) were vertically placed in a furnace. The furnace temperature was regulated according to the standard cellulosic fire curve described in ISO 834-2 [47] by a control unit of a gas burner fed with a propane–butane mixture. The temperature values, as well as the temperature of the steel substrates, were tracked using K-type thermocouples and continuously recorded. Each test continued until the steel temperature reached 350 °C (the critical temperature value measured on the backside of the tested sample). Three coated panels were tested, and the arithmetic mean was calculated.

#### 2.4.7. Corrosion Resistance

The cuts were made on the coating in accordance with ISO 17872 [48] in the shape of a letter X, through the coating to the metal using a special knife so that the ends of the incisions were 20 mm from the edge of the tile. The samples were put in a 3.5% NaCl solution at 35 °C for 650 h and 720 h in order to test the corrosion resistance in accordance with ISO 2812-1 [49]. The degree of delamination and corrosion around the scratches was evaluated according to the ISO 4628-8 [50] standard. Three coated panels were tested, and the arithmetic mean was calculated.

## 3. Results and Discussion

### 3.1. Characteristics of the Powder Coatings Composition and Manufacturing Process

The polyurethane powder coatings containing 1.5 wt%, 2.5 wt%, and 5 wt% of the cyclophosphazene modifier were obtained as a result of the addition of hexakis(4-(hydroxymethyl)phenoxy)cyclotriphosphazene to the mixture containing polyester resin and special additives for powder systems. The composition of the mixture is presented in Table 1. Chemical modification was used to modify standard polyurethane powder coatings. The amount of cross-linking agent was chosen such that it could react with the hydroxyl groups of the resin and phosphazene. During the cross-linking process, the hydroxyl groups derived from the modifier reacted with the isocyanate groups of the polyisocyanate, which allowed them to be chemically incorporated into the coating structure. The modifier was added to the mixture at the premilling stage. During the extrusion of the modified powder mixture, no negative effect of the modifier on the homogenization of the powder coating components was observed. Due to the high melting point of the modifier, a higher curing temperature (220 °C) was used for standard polyurethane powder coatings (180 °C). After 15 min of curing in the oven, the received coatings were characterized by a smooth and transparent surface, and no defects were also observed. 

### 3.2. Powder Coatings Performance Properties

After the curing process, a polymerization test was performed to verify whether the coating was cross-linked. Coatings were qualified at level 4 according to the polymerization test, which is the highest on this scale. After thirty double rubs, the coatings did not show a perceptible change and could not be scratched with a fingernail. According to the Qualicoat requirements, this is a very satisfactory result. The investigated powder coating exhibits perfect chemical resistance to methyl ethyl ketone. To investigate the influence of the phosphazene modifier on the mechanical properties of the powder coatings obtained, their roughness, adhesion to the steel surface, hardness, gloss, cupping, and scratch resistance were examined. The results of the mechanical properties of the cured powder coatings are shown in Table 2. The modification of polyurethane coatings with cyclophosphazene did not significantly influence the mechanical properties of the polyurethane powder coatings. The values of properties, such as adhesion to the steel substrate, hardness, cupping, and scratch resistance, remained at the same level as those of the reference sample. For example, on a scale of 0–5 (0—best, 5—worst), the obtained coatings were characterized by the highest degree in this scale. The lack of a visible effect of the cyclophosphazene modifier on the mechanical properties of the coating, such as scratch resistance, may be due to a small difference in the energy of bonds occurring in the cyclophosphazene ring (P-N = 209 kJ/mol and P-O = 351 kJ/mol) and polyurethane (N-O = 201 kJ/mol, C-N = 305 kJ/mol, C-O = 358 kJ/mol). The properties, such as hardness and cupping, can be influenced by the presence of aromatic substituents in the structure of the phosphazene modifier, which should increase these parameters. However, probably more important here is the flexible “articulated” action of the ether oxygen atom that connects these aromatic rings with the phosphazene ring. An increase in the gloss value from 101.6 GU to 107.1 GU as the modifier content in the obtained cured coatings increased. The observed increase in gloss indicates the very good compatibility of the modifier with the polyurethane matrix, which may be due to the hydrophilic–hydrophobic structure of both compounds. With the increase in the modifier content, the water contact angle decreased from 84.9 to 83.0 deg. The reduced contact angle for water is a consequence of the hydrophilic structure of the cyclophosphazene modifier.

### 3.3. Phosphorus Distribution in the Cured Coatings

**Figure 2:**
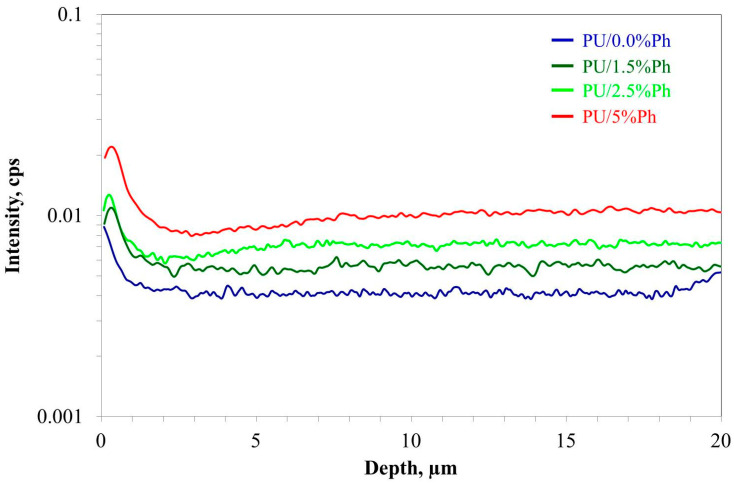
GD-OES depth profiles illustrating the intensity of light emitted by phosphorus as a function of depth.

The GD-OES depth profiles that illustrate the intensities of light emitted by phosphorus as a function of depth are shown in Figure 2. The concentration of phosphorus in powder coatings was qualitatively estimated. The intensity of light emission by phosphorus from the PU/0.0%Ph sample showed a value at the background level and correlated with a phosphorus concentration of zero. Coatings containing the cyclophosphazene modifier showed a higher intensity of light emission by phosphorus, which increases with the increasing cyclophosphazene content in the sample. This indicates the presence of phosphazene in these coatings, the content of which increases by the order of PU/1.5%Ph, PU/2.5%Ph and PU PU/5%Ph. The phosphorus content on the surface of the coatings slightly increases, while at a depth above 2 μm, it remains constant. The observed increase in phosphorus concentration at the coating surface may be related to the migration of phosphazene-derived segments toward the coating surface. This migration is a result of the thermodynamic incompatibility of phosphazene-derived segments with polyurethane segments and an increase in interfacial energy. As a result of the migration of these incompatible segments to the surface, the interfacial energy in the coating is reduced. This migration is inhibited by chemical bonds formed between the cyclotriphosphazene hydroxyl groups and polyisocyanate during the coating cross-linking process. A similar process was observed in our earlier research on polyurethane powder coatings modified with polysiloxanes or fluoropolyols [51].

### 3.4. Thermal Stability

**Figure 3:**
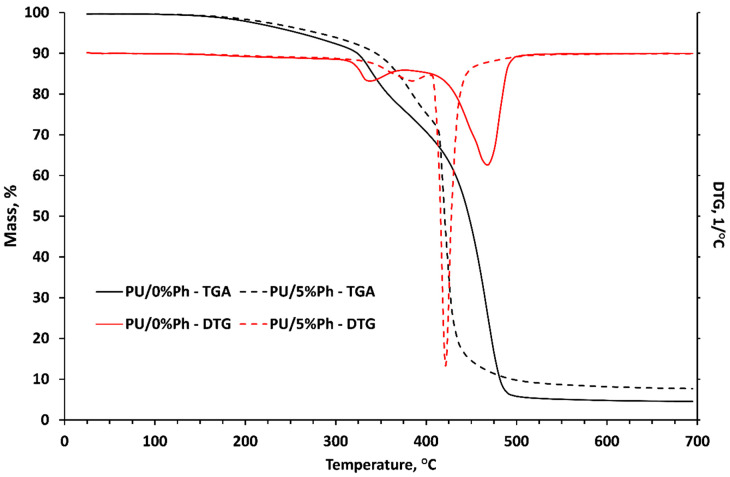
TG and DTG curves of samples PU/0.0%Ph and PU/5%Ph.

Thermogravimetric analysis was performed to investigate the effect of cyclophosphazene on the thermal stability of fire-protective powder coatings. The TG and DTG curves of the reference PU sample and a sample that contained 5% cyclophosphazene are presented in Figure 3.

The unmodified polyurethane coating decomposes in two stages in the temperature range of 320–520 °C. The first step occurs at the temperature of the maximum mass loss rate of T_max1_ = 337 °C and is related to the scission of the urethane bond, while the second step (T_max2_ = 467 °C) is associated with the degradation of the polyester segments. The mass losses in the first and second steps amount to 23.8% and 71.2%, respectively. The temperature of 5% mass loss is 258 °C. A similar decomposition pattern is observed for the modified sample, but the thermal decomposition range is narrower (320–450 °C). The modification of the sample resulted in an increase in temperature of 5% of the mass loss by approximately 22 °C, which confirms the positive impact of cyclophosphazene on the thermal stability of the coating. That increase in thermal stability may also result from the increase in cross-link density as a result of the incorporation of hexafunctional cyclophosphazene. The T_max_ of the first degradation stage is higher (T_max1_ = 385 °C), but T_max2_ is lower, 420 °C compared to the T_max_ of the unmodified sample. This phenomenon may be related to the catalytic effect of phosphoric and polyphosphoric acids formed during the decomposition of phosphazene on the degradation of polyester segments, as reported in Ref. [52]. Moreover, the decomposition of a phosphazene-modified sample occurs in a narrower temperature range than that of an unmodified sample, and it ends at a lower temperature value. Thus, the formation of solid residue after degradation, which acts as an insulating layer and prevents further degradation, occurs earlier. Additionally, the amount of char yield, i.e., 8%, is higher for the modified sample compared to 5% for pristine polyurethane. The higher char yield enhances the fire retardancy of the polyurethane coatings. 

### 3.5. Thermal Protection

**Figure 4:**
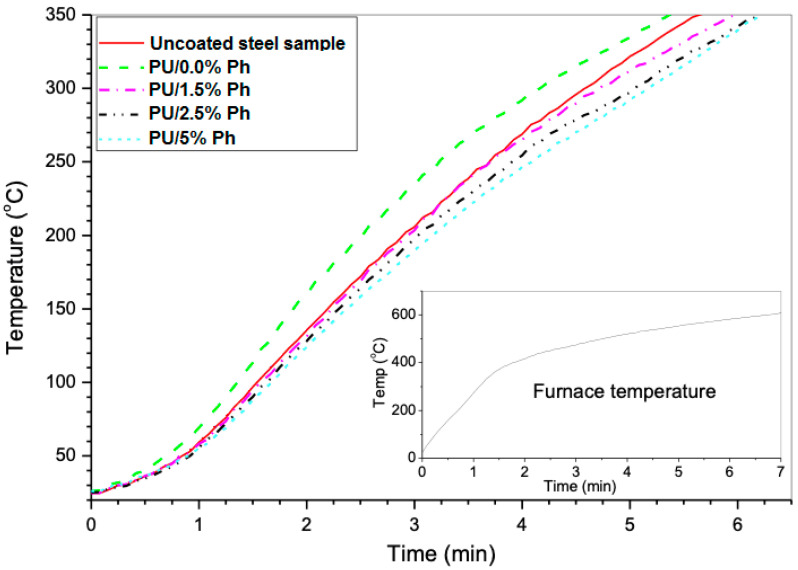
Temperature–time profiles for coated steel samples exposed to standard cellulose fire heating in the furnace.

**Figure 5:**
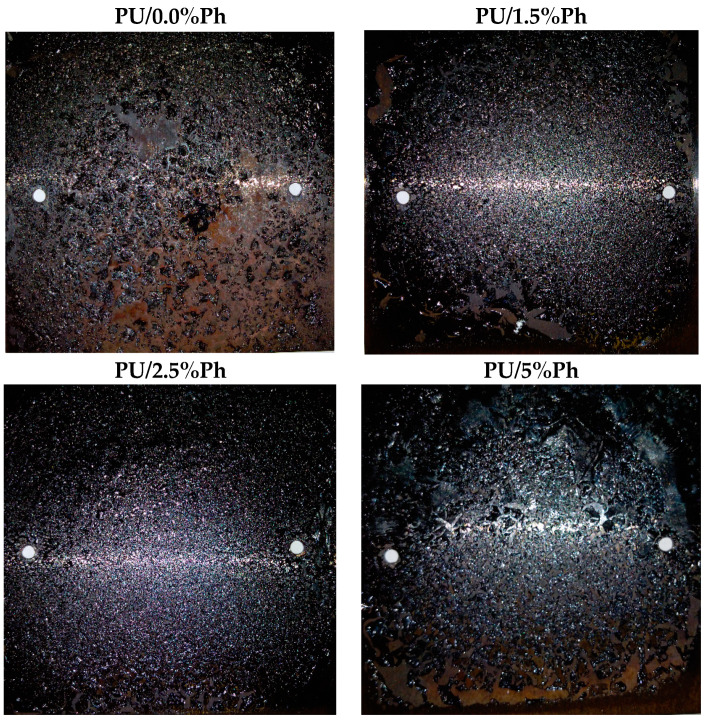
Photographs of powder coatings after the furnace test.

The furnace test was used to study the thermal-resistant character of the modified PU powder coatings. The profiles of temperature changes (of the coated steel substrates) in relation to the test duration are shown in Figure 4, while the photographs of the coatings, taken after the fire test, are presented in Figure 5. Detailed time values at which the coating reached a temperature of 350 °C are shown in Table 3. 

For the uncoated sample and the sample coated with the reference paint, the increase in the steel substrate temperature was greater than for the plates painted with the cyclotriphosphazene-containing coatings. It was shown that the addition of hexakis(4-(hydroxymethyl)phenoxy)cyclotriphosphazene to the polyurethane powder coatings improved their thermal resistance. The uncoated steel sample reached 350 °C after 5 min 40 s of heating, while the PU/1.5%Ph sample reached this temperature 20 s later. Increasing the modifier amount in the coating by 1% increased the time by another 12 s (2.5%Ph sample). An additional increase in the additive concentration in the coating did not significantly increase the time required to reach the specified temperature of the steel substrate. 

Differences in the time at which the coating reached a temperature of 350 °C for the tested samples were not significant due to the fact that the tested coatings had a small thickness in the range of 71.9–73.6 µm. A greater difference in the time needed to reach 350 °C is possible when the coating is thicker. However, the thickness of the tested powder coatings complies with the technical requirements of Qualicoat [28]. In the case of powder coatings, as opposed to liquid coatings, increasing their thickness results in the deterioration of mechanical properties.

Figure 5 shows the surface of the coatings after the furnace test, which had charred and created a heat insulation barrier between the unburned parts. 

As confirmed by TG studies, the amount of char increases with the increasing cyclophosphazene content. Moreover, the charring of the unmodified sample is due to its loose structure, whereas the carboceneous phosphazene-modifed coatings are significantly more compact. The tighter char structure is due to the formation of phosphoric acid, metaphosphoric acid, and polyphosphoric acid during the decomposition of HHCPC, which remains in the condensed phase [23]. The liquid or semi-liquid states of these compounds cause an increase in volume and seal the char layer, which is a better protective shield to prevent the flammable volatiles from penetrating the surface of the coating into the flame zone during the process of burning. The surface morphology of the char residue after the furnace test shows an alveolate structure, which indicates that non-flammable gas, such as water, was released during combustion, which was also observed by Xu et al. during the decomposition of HHPCP [23]. Non-flammable gas and flammable volatiles, such as toluene, benzene, and the benzene fragments generated and volatilized into the gaseous phase, cannot easily penetrate the compact char layer into the flame zone during the burning process, which contributes to slowing down this process. In addition, a thicker and more compact layer of char slows down the heat transfer from the flame to the unburned part more effectively, protecting the substrate than the loose and thinner layer formed by reference coatings. 

### 3.6. Corrosion Properties

An examination and explanation of the anticorrosion effect of HHPCP-modified coatings is a novelty of this work. To assess the protective properties of the coating against corrosion, immersion tests were performed. The coatings on the steel substrates were placed in a 3.5% NaCl solution at 35 °C for 650 and 720 h according to ISO 2812-1. After immersion tests, the samples were cleaned, the delaminated coating was removed, and coating damage was evaluated according to ISO 4628 1-10 [53]. According to ISO 4628-8, the degree of coating delamination was calculated using Formula (1), and the degree of corrosion around the scratches occurred according to Formula (2):

(1) $$d=\frac{A_{d}-A_{l}}{2}\cdot \frac{1}{l}$$

where:

*A_d_*—delamination area including scratch area, [mm^2^];
*A_l_*—scratch area, [mm^2^], 30 mm^2^;
*l*—scratch length, [mm], 100 mm.

(2) $$c=\frac{w_{c}-w}{2}$$

where

*w_c_*—average width of the corrosion zone, [mm];
*w*—width of the original crack, [mm], 0.3 mm.

**Figure 6:**
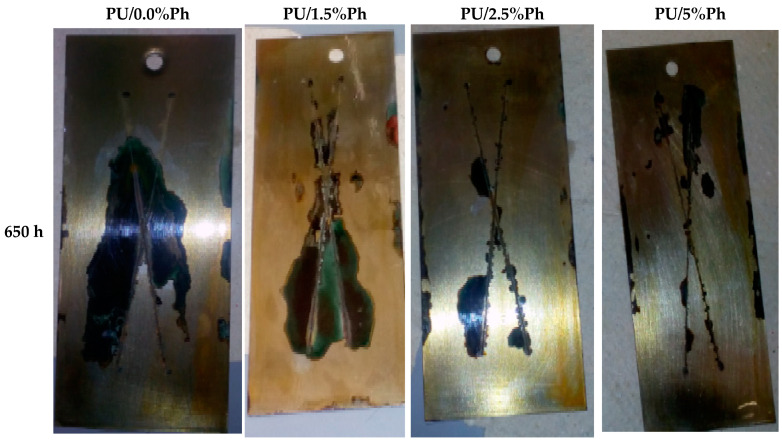

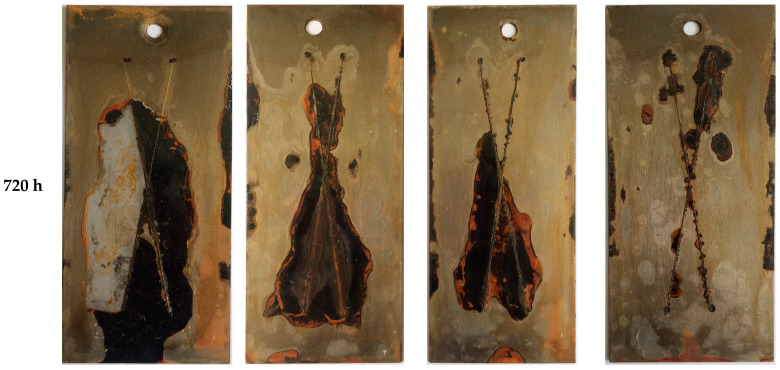
Photographs of the coatings taken after 650 and 720 h of exposure to a 3.5% NaCl solution.

The corrosion protection performance of the various coatings containing different amounts of the cyclophosphazene modifier is shown in Table 4, and photographs of modified powder coatings after 650 and 720 h of exposure to a 3.5% NaCl solution are shown in Figure 6. The first measurement was taken after 650 h.

Based on the results obtained, it was found that the sample containing 5% cyclophosphazene revealed the best anticorrosive properties. No delamination occurred around the scratches, and the degree of corrosion around the scratches was the lowest in the case of these samples. The largest delamination and corrosion around the scratches was observed in the case of a reference sample. The subsequent measurement was made after 720 h. After this time, delamination occurred for each coating sample. However, the lowest degree of delamination around the scratches was observed in the sample containing 5% cyclophosphazene. Delamination below d < 5 mm indicates a positive test result. For each coating sample, the degree of corrosion around the scratches increased. The results confirmed that a higher amount of modifier improved the anticorrosion properties of the coating. Due to the chemical structure of the phosphazene ring (three conjugated double bonds and free electron pairs on nitrogen atoms), HHPCP can show electroactive properties, protecting the steel substrate against corrosion not only by a barrier but also by anodizing, as confirmed by Tansug et al. in the case of coatings with the addition of electroactive polymers such as polypyrrole or polyaniline [54]. The P heteroatoms contained in the phosphazene ring can adsorb on the metal surface in two ways: by physical adsorption or chemisorption, protecting it against corrosion [26]. In addition, this effect is enhanced due to the higher concentration of phosphazene-derived segments at the coating surface, which was confirmed by the GD-OES technique. The corrosion protection of the coating modified with HHPCP is related to the barrier effect of the polyurethane binder. HHCPC has a rather polar structure, as evidenced by the lack of increase in the water contact angle, which does not have a positive effect on increasing barrier protection.

## 4. Conclusions

As part of this study, the influence of the hexakis(4-(hydroxymethyl)phenoxy)cyclotriphosphazene modifier on the properties of PU powder coatings was investigated, which was the novelty of this work. The applied modification by the chemical incorporation of the modifier into the coating structure prevents the uncontrolled release of the modifier during exploitation. The release of the modifier is impossible due to permanent chemical bonding. When the modifier forms only a physical mixture without chemical bonds, it is easy to release the modifier into the environment during exploitation, leading to the deterioration of the coating performance.

We proved that HHPCP improved the thermal and anticorrosion resistance of the coatings. The HHPCP-modified coatings revealed increased fire protection compared to that of the unmodified sample; however, due to the technical requirements of the powder coating thickness, the improvement was not significant. The appropriate thickness of the powder coating is necessary so that such properties of the coating, such as gloss, roughness, hardness, scratch resistance, cupping, water contact angle, and adhesion to steel, do not deteriorate. These parameters were maintained at a very high level, especially adhesion to the substrate, which is crucial in ensuring adequate anticorrosion protection of the steel.

The properties of cyclotriphosphazene-modified coatings are highly advantageous when they are used to provide protection for steel or other materials against different environmental influences, especially corrosion. Due to these improved properties, modified coatings with success can be used to protect materials used outdoors, especially susceptible to corrosion, such as steel exploited in highly corrosive environments (e.g., corrosivity category C4 and C5). This property extends the service life of treated materials compared to those which are protected with unmodified coatings.

Based on this research, it can be concluded that the HHPCP modifier can be successfully used as a green alternative to obtain environmentally friendly halogen- and zinc-free powder coatings with increased thermal stability and corrosion resistance. The optimal content of the phosphazene modifier in the powder coating should be 5 wt.%.

## Figures and Tables

**Table 1:** Formulations of powder coatings.

Materials	Vestagon B 1530 [%]	Sirales PE 6110 [%]	Phosphazene Modifier [%]	Benzoin [%]	Resiflow PV 88 [%]
Coating Symbol					
PU/0.0%Ph	17.75	81.50	0.00	0.25	0.50
PU/1.5%Ph	19.23	78.72	1.50	0.25	0.50
PU/2.5%Ph	20.55	76.20	2.50	0.25	0.50
PU/5%Ph	20.25	74.00	5.00	0.25	0.50

**Table 2:** Specifications of the powder coating properties.

Symbol of Coating		PU/0.0%Ph	PU/1.5%Ph	PU/2.5%Ph	PU/5%Ph
Roughness ISO 12085	Ra Rz	1.20 ± 0.02 4.30 ± 0.20	1.09 ± 0.02 3.21 ± 0.18	1.12 ± 0.03 3.33 ± 0.17	0.97 ± 0.05 2.40 ± 0.14
Gloss for the angle of 60° ISO 2813	GU	101.6 ± 1.3	106.2 ± 1.5	105.9 ± 1.4	107.1 ± 1.5
Adhesion to the steel surface ISO 2409	0—best 5—worst	0	0	0	0
Hardness ISO 1522	-	0.808 ± 0.002	0.806 ± 0.003	0.806 ± 0.003	0.807 ± 0.003
Cupping ISO 1520	mm	10.66 ± 0.10	10.63 ± 0.07	10.63 ± 0.06	10.62 ± 0.09
Scratch resistance ISO 1518	g	350	350	350	350
Water contact angle EN 828	deg	84.9 ± 2.3	84.7 ± 2.5	84.0 ± 2.1	83.0 ± 2.1

**Table 3:** Final time of reaching temperature of 350 °C.

**Sample**	**Time**	**Coating Thickness, µm**
Uncoated steel sample	5 min 40 s ± 1 s	-
PU/0.0%Ph	5 min 25 s ± 1 s	73.6 ± 1.2
PU/1.5%Ph	6 min ± 1 s	71.9 ± 1.0
PU/2.5%Ph	6 min 12 s ± 1 s	73.2 ± 1.1
PU/5%Ph	6 min 13 s ± 1 s	72.1 ± 1.0

**Table 4:** Characteristics of the powder coating damage after 650 and 720 h of exposure to a 3.5% NaCl solution.

Symbol of Coating	Degree of Delamination around the Scratches, mm		Degree of Corrosion, around the Scratches, mm	
	650 h	720 h	650 h	720 h
PU/0.0%Ph	6.80 ± 0.05	8.20 ± 0.04	2.63 ± 0.02	3.75 ± 0.02
PU/1.5%Ph	no damages	6.23 ± 0.05	1.08 ± 0.02	1.80 ± 0.02
PU/2.5%Ph	no damages	6.85 ± 0.03	0.52 ± 0.01	1.73 ± 0.01
PU/5%Ph	no damages	3.05 ± 0.02	0.10 ± 0.01	0.43 ± 0.01

## Data Availability

The original data presented in the study are included in the article. Additional raw data supporting the conclusions of this article will be made available by the authors on request.

## Supplementary Materials

The following supporting information can be downloaded at: https://www.mdpi.com/article/10.3390/ma17112672/s1, Figure S1: FT-IR spectrum of Hexakis(4-formylphenoxy)cyclotriphosphazene (2); Figure S2: ^1^H-NMR spectrum of Hexakis(4-formylphenoxy)cyclotriphosphazene (2) in CDCl_3_; Figure S3: ^13^C NMR spectrum of Hexakis(4-formylphenoxy)cyclotriphosphazene (2) in CDCl_3_; Figure S4: ^31^P NMR spectrum of Hexakis(4-formylphenoxy)cyclotriphosphazene (2) in CDCl_3_; Figure S5: FT-IR spectrum of Hexakis(4-(hydroxymethyl)phenoxy)cyclotriphosphazene (3); Figure S6: ^1^H NMR spectrum of Hexakis(4-(hydroxymethyl)phenoxy)cyclotriphosphazene (3) in DMSO-d6; Figure S7: ^13^C NMR spectrum of Hexakis(4-(hydroxymethyl)phenoxy)cyclotriphosphazene (3) in DMSO-d6; Figure S8: ^31^P NMR spectrum of Hexakis(4-(hydroxymethyl)phenoxy)cyclotriphosphazene (3) in DMSO-d6.
