# Supplementary material for: Hexakis[p-(hydroxymethyl)phenoxy]cyclotriphosphazene as an Environmentally Friendly Modifier for Polyurethane Powder Coatings with Increased Thermal Stability and Corrosion Resistance
Source: Materials (Basel). 2024 Jun 1;17(11):2672. doi: 10.3390/ma17112672 (PMC11174046; doi:10.3390/ma17112672)
Supplement: Supplementary file 1 [file materials-17-02672-s001.zip › materials-2987668-supplementary.pdf]

Supplementary Materials

Hexakis[p-(hydroxymethyl)phenoxy]cyclotriphosphazene as an Environmentally Friendly Modifier for Polyurethane Powder Coatings with Increased Thermal Stability and Corrosion Resistance

Barbara Pilch-Pitera 1,*, Dominika Czachor-Jadacka 1, Łukasz Byczyński 1, Michał Dutkiewicz 2, Rafał Januszewski 3,4, Krzysztof Kowalczyk 5, Wojciech J. Nowak 6 and Katarzyna Pojnar 7

1 Faculty of Chemistry, Department of Polymers and Biopolymers, Rzeszow University of Technology, al. Powstańców Warszawy 6, 35-959 Rzeszow, Poland
2 Poznan Science and Technology Park, Adam Mickiewicz University Foundation, Rubież 46, 61-612 Poznan, Poland
3 Faculty of Chemistry, Adam Mickiewicz University in Poznan, Umultowska 89B, ul. Uniwersytetu Poznańskiego 8, 61-614 Poznan, Poland
4 Center for Advanced Technology, Adam Mickiewicz University in Poznan, ul. Uniwersytetu Poznańskiego 10, 61-614 Poznan, Poland
5 Faculty of Chemical Technology and Engineering, Polymer Institute, West Pomeranian University of Technology in Szczecin, ul. Pułaskiego 10, 70-322 Szczecin, Poland
6 Faculty of Mechanical Engineering and Aeronautics, Department of Materials Science, Rzeszow University of Technology, al. Powstańców Warszawy 8, 35-959 Rzeszow, Poland
7 Doctoral School of Engineering and Technical Sciences at the Rzeszow University of Technology, al. Powstańców Warszawy 12, 35-959 Rzeszow, Poland

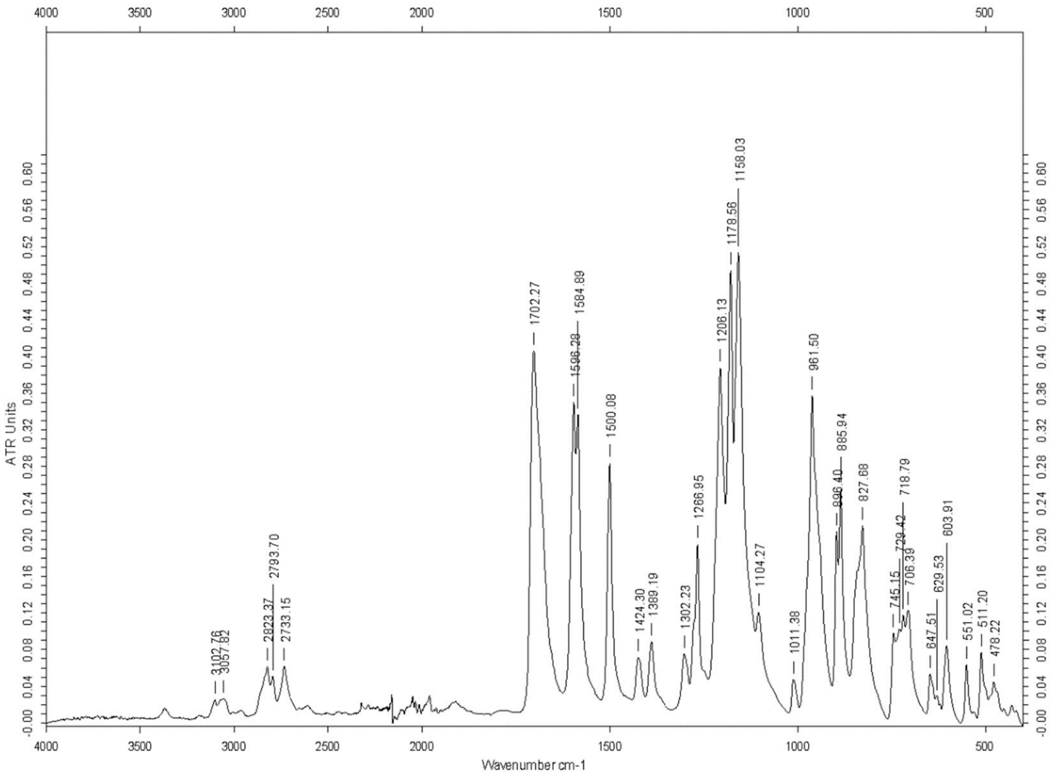

Figure S1. FT-IR spectrum of Hexakis(4-formylphenoxy)cyclotriphosphazene (2).

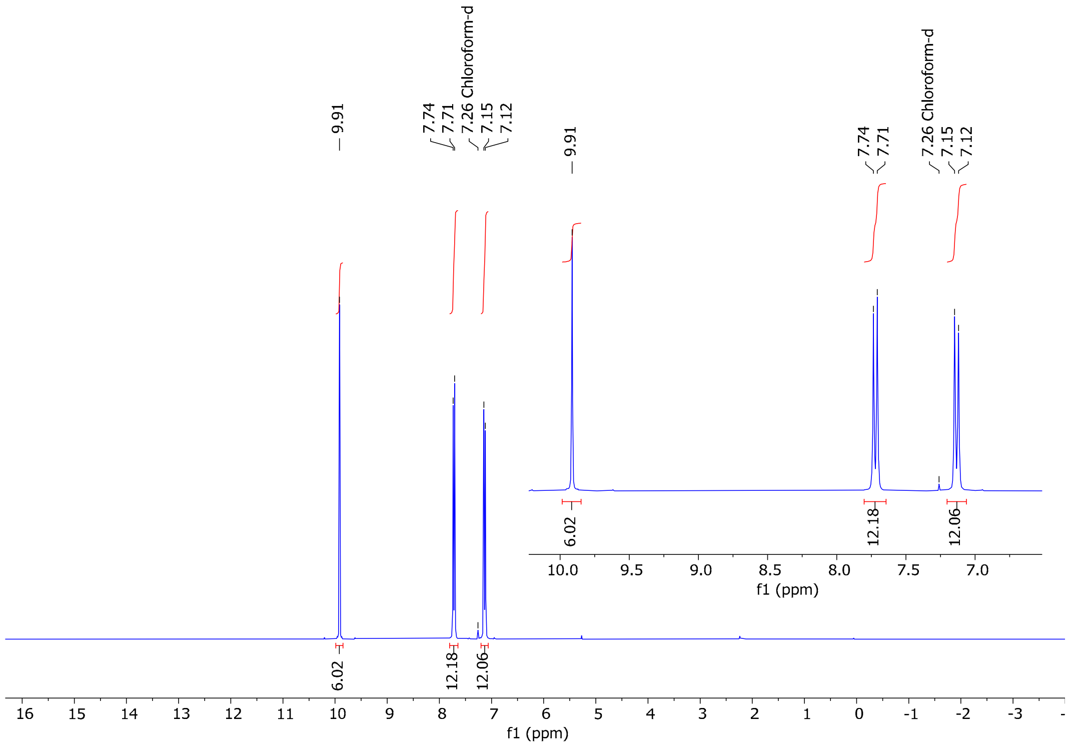

Figure S2. ^{1}H NMR spectrum of Hexakis(4-formylphenoxy)cyclotriphosphazene (2) in $CDCl_3$.

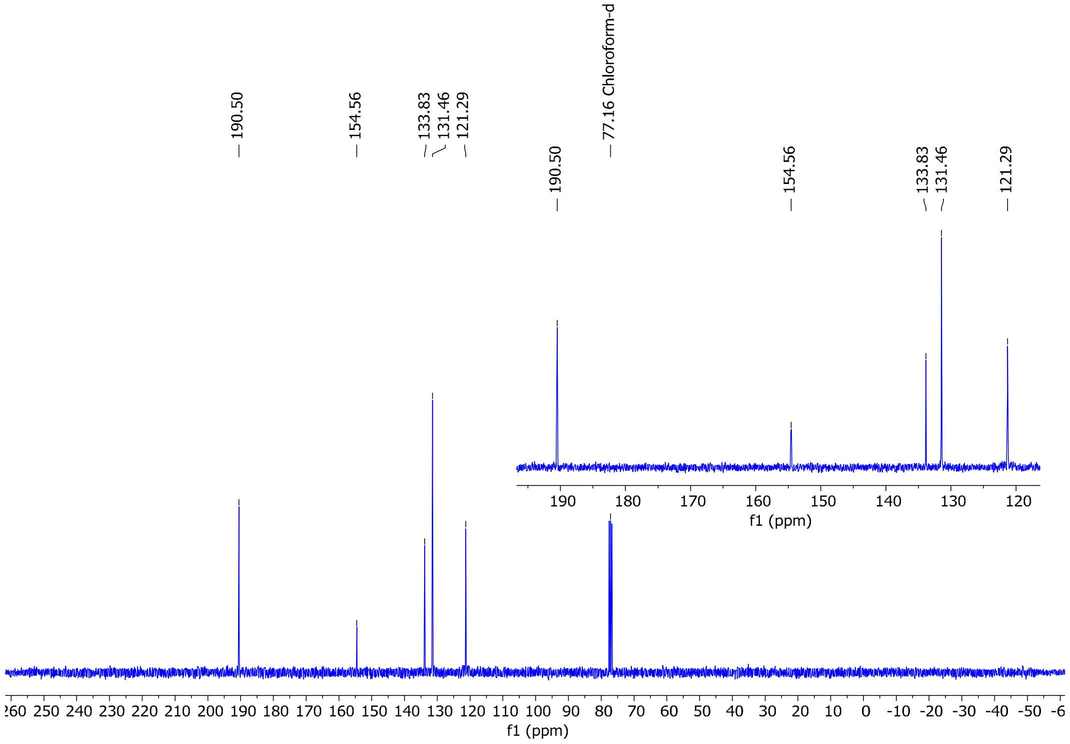

Figure S3. ^{13}C NMR spectrum of Hexakis(4-formylphenoxy)cyclotriphosphazene (2) in $CDCl_3$.

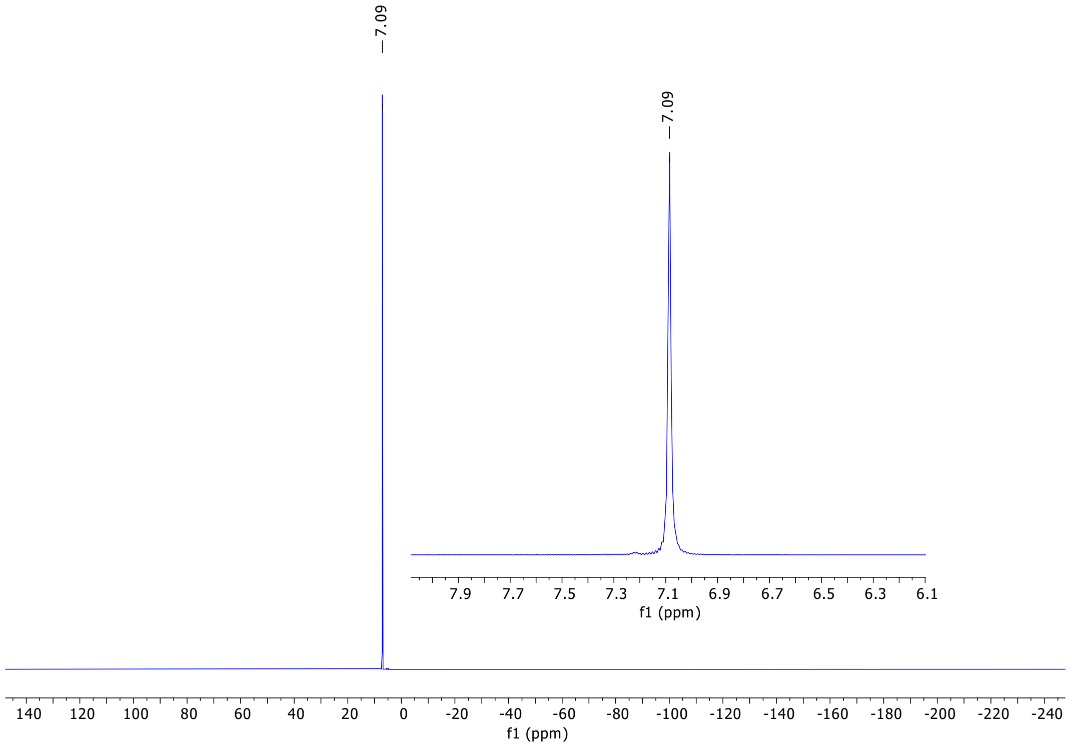

Figure S4. ^{31}P NMR spectrum of Hexakis(4-formylphenoxy)cyclotriphosphazene (2) in $CDCl_3$.

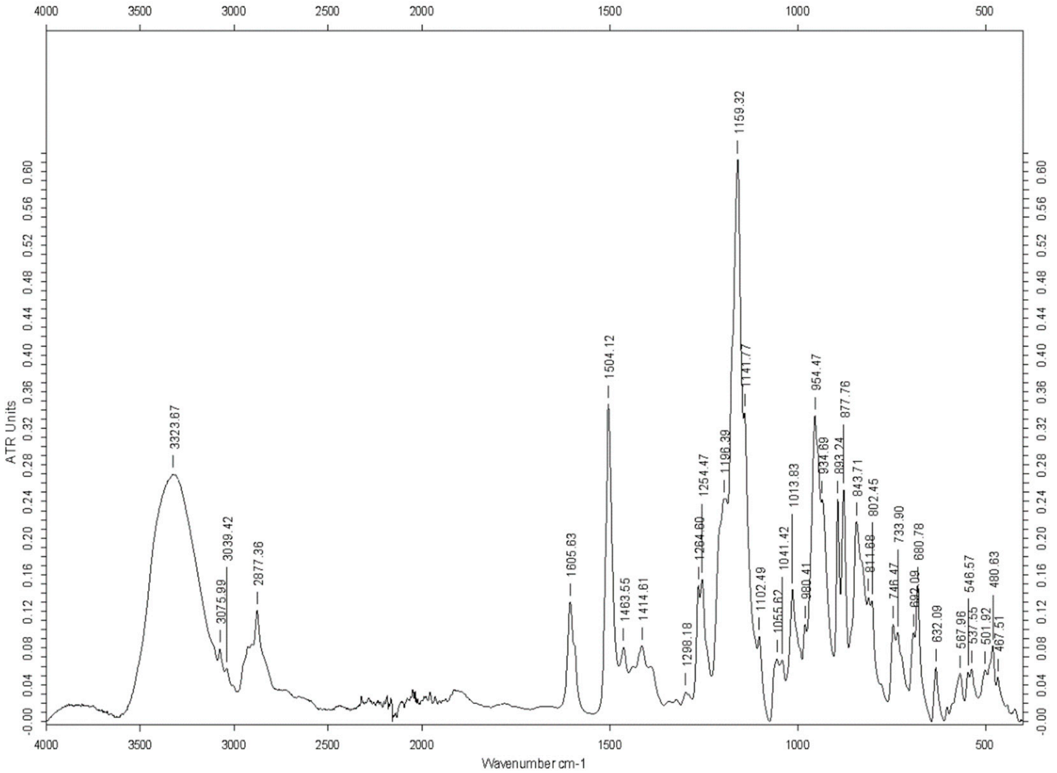

Figure S5. FT-IR spectrum of Hexakis(4-(hydroxymethyl)phenoxy)cyclotriphosphazene (3).

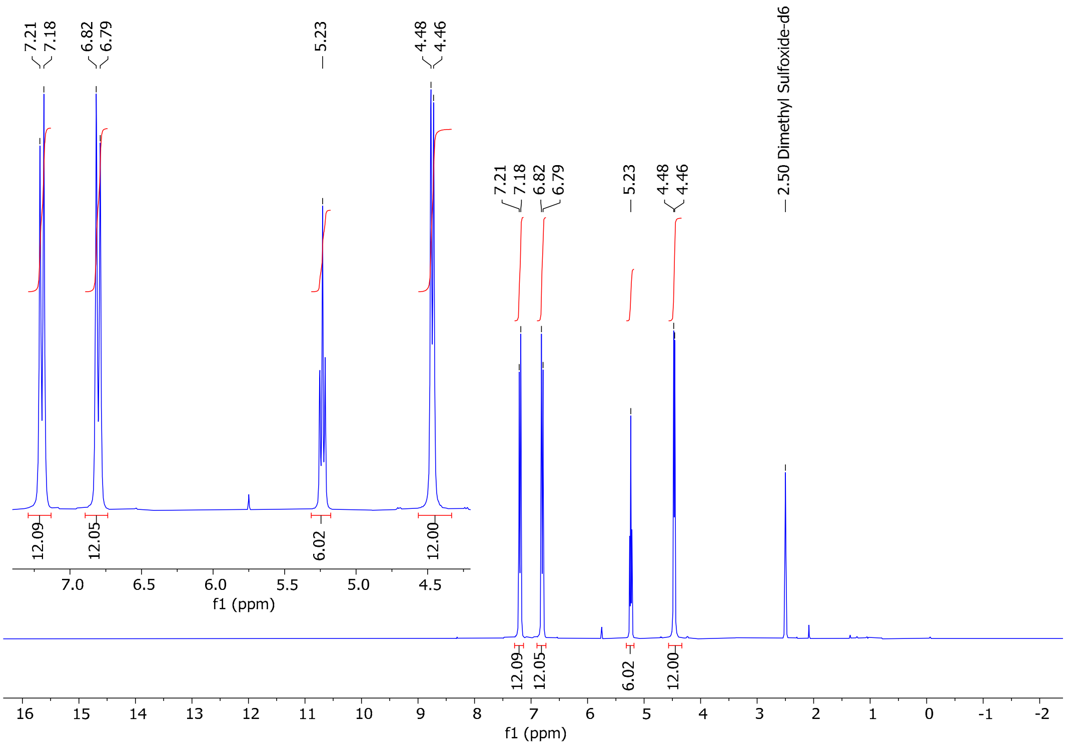

Figure S6. ^{1}H NMR spectrum of Hexakis(4-(hydroxymethyl)phenoxy)cyclotriphosphazene (3) in DMSO-d6.

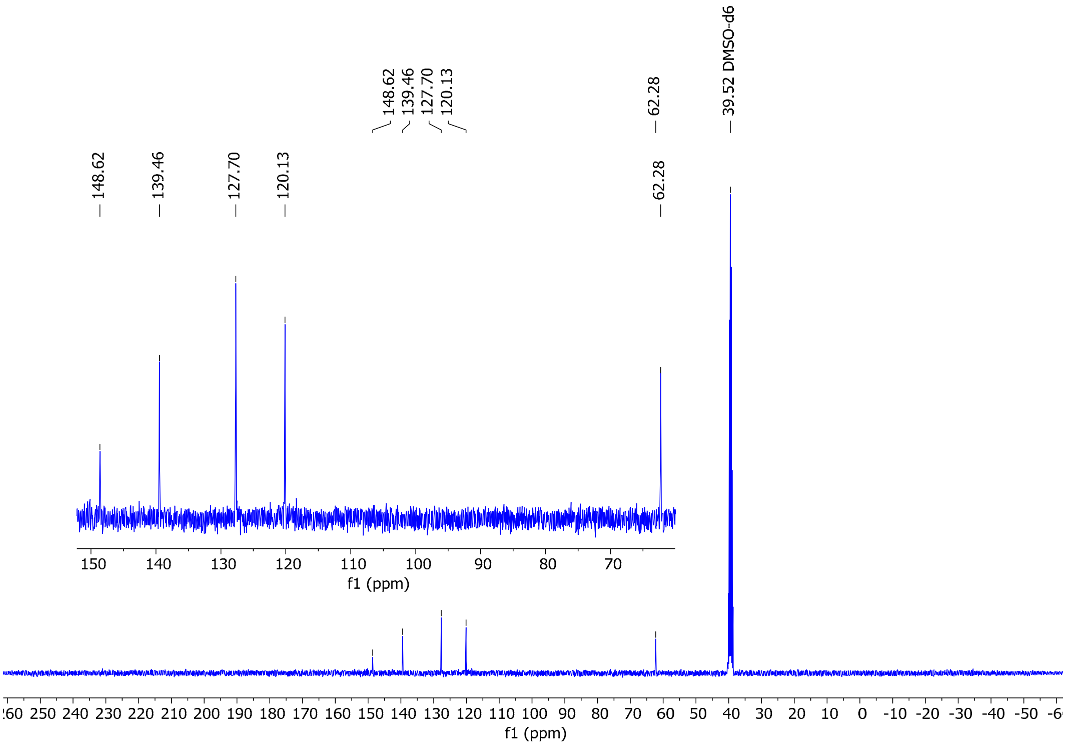

Figure S7. ^{13}C NMR spectrum of Hexakis(4-(hydroxymethyl)phenoxy)cyclotriphosphazene (3) in DMSO-d6.

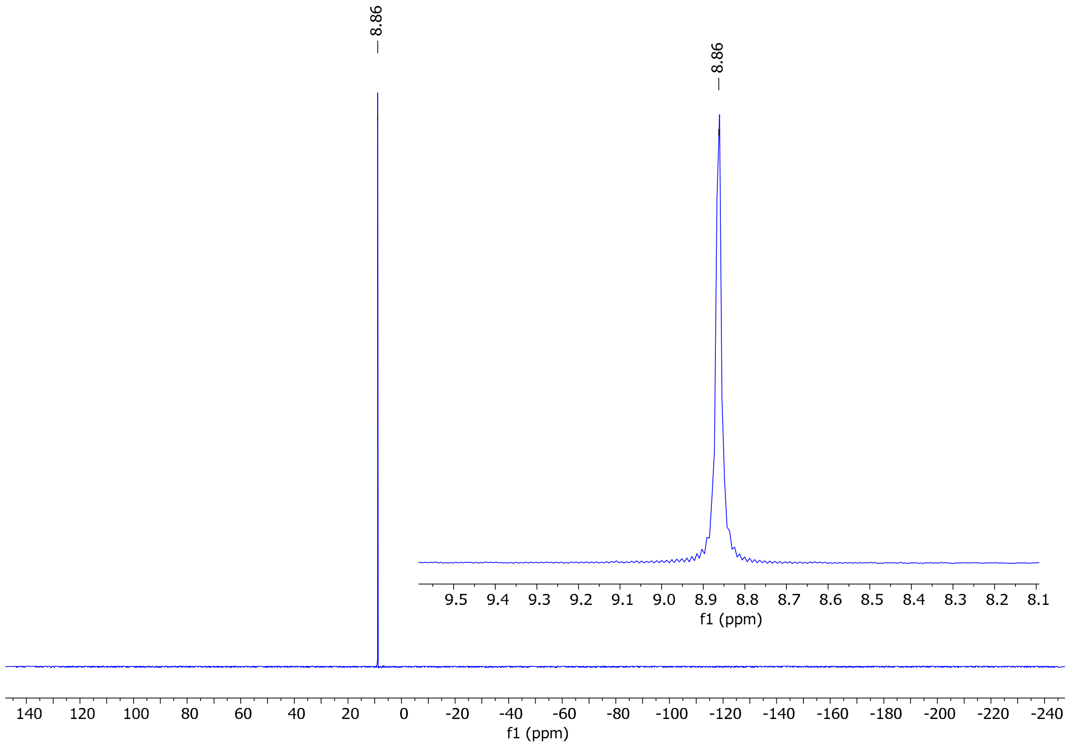

Figure S8. ^{31}P NMR spectrum of Hexakis(4-(hydroxymethyl)phenoxy)cyclotriphosphazene (3) in DMSO-d6.
